# Novel rapid immunohistochemistry using an alternating current electric field identifies Rac and Cdc42 activation in human colon cancer FFPE tissues

**DOI:** 10.1038/s41598-022-05892-7

**Published:** 2022-02-02

**Authors:** Masumi Tsuda, Runa Horio, Lei Wang, Tomoko Takenami, Jun Moriya, Jun Suzuka, Hirokazu Sugino, Zenichi Tanei, Mishie Tanino, Shinya Tanaka

**Affiliations:** 1 Department of Cancer Pathology, Faculty of Medicine, Hokkaido University, N15, W7, Kita-ku, Sapporo, 060-8638 Japan; 2 Institute for Chemical Reaction Design and Discovery (WPI-ICReDD), Hokkaido University, Sapporo, Japan; 3 Global Station for Soft Matter, Global Institution for Collaborative Research and Education, Hokkaido University, Sapporo, Japan; 4 Present Address: Department of Diagnostic Pathology, Asahikawa Medical University Hospital, Asahikawa, Japan

**Keywords:** Biotechnology, Assay systems

## Abstract

It is important to determine the activation status of Rac and Cdc42 in cancer tissues for the prediction of metastasis and patient prognosis. However, it has been impossible to detect their spatial activation on formalin-fixed paraffin embedded (FFPE) surgical specimens thus far. Here, we established a novel detection technique for activated Rac/Cdc42 in human colon cancer FFPE tissues by using a p21-activated kinase (PAK)-Rac binding domain (RBD) detection probe fused with glutathione S-transferase (GST), designated GST-PAK-RBD, and novel rapid-immunohistochemistry (R-IHC) systems using noncontact alterating-current electric field mixing, although there is a technical limitation in that it may not distinguish between Rac members and Cdc42. In 50 cases of colon cancer, various activation patterns of Rac/Cdc42 were observed, which were designated plasma membrane, cytoplasm, mixed pattern, and polarized distribution. The activity was striking in the invasive fronts of tumors and significantly correlated with tumor invasion properties evaluated by TNM classification. Of note, in tissue microarray (TMA) samples, 29 of 33 cases demonstrated higher Rac1/Cdc42 activity in the tumor area than the corresponding normal mucosa. In addition, positive correlations were detected between Rac/Cdc42 activity and clinicopathological factors such as venous and lymphatic vessel invasion. These results suggest that understanding Rac and Cdc42 activations in cancer tissues would be valuable as an option for molecular therapy as personalized medicine.

## Introduction

Tumor invasion and metastasis are prognostic factors in various cancer patients. The potential of tumor cell migration and invasion seems to be individually distinct, as evaluated by the expression of epithelial-mesenchymal transition (EMT) markers such as Snail, Slug, N-cadherin, and Vimentin^1^. Although an understanding of tumor migration and invasion capabilities would be indispensable for predicting metastasis and patient prognosis, to date, only microscopic examination using H&E staining has been performed on surgical specimens of human cancer formalin-fixed paraffin embedded (FFPE) tissues^2^.

Rac and Cdc42 are key regulators that promote cell motility and are associated with tumor invasion and metastasis. Small GTPases comprise several families, including the Ras, Rho, and Rab families. The Rho family contains three well-characterized GTPases, RhoA, Rac1, and Cdc42, which cooperatively regulate the assembly and organization of the actin cytoskeleton. RhoA participates in the formation of stress fibers and focal adhesion complexes, Rac1 promotes the assembly of a meshwork of actin filaments to produce lamellipodia and membrane ruffles, and Cdc42 leads to the formation of filopodia and actin-rich surface protrusions^3^.

Small GTPases are activated by guanine nucleotide exchange factor (GEF) binding to GTP but inactivated by GTPase-activating protein (GAP). The GTP-bound form of small GTPases interacts with distinct effector molecules to execute their cellular function^4,5^. Rac1/Cdc42-GTP bind to p21-activated kinase (PAK) and regulate various cellular functions, such as cytoskeleton dynamics and gene expression, leading to cell movement, migration, and proliferation^6–8^. Activated Rho family proteins contribute to promoting the metastasis of tumor cells by disrupting epithelial sheet organization, increasing cell motility, and promoting the degradation of the extracellular matrix^9^. Rac1b, a splice variant of Rac1 showing constitutively active GTPase, was preferentially expressed in patients with colon and breast cancer^10–12^. In addition, overexpression and gene amplification of PAK1 have been shown in various cancers, including breast, colon, ovarian, bladder, brain, and pancreatic cancers^13^, suggesting a pivotal role of active Rac1/Cdc42 in human carcinogenesis. Thus, it is important to evaluate the spatial activation of intrinsic Rac1/Cdc42 in tumor tissues.

The efficient detection method of small G-protein by using FFPE tissues has not yet been established due to the low binding frequency of the detection probe. In the present study, we developed a novel detection technique for activated Rac/Cdc42 in human cancer FFPE tissues using a recently developed rapid immunohistochemistry (R-IHC) device, in which the antigen–antibody reaction can be facilitated up to 1,200-fold by alternating current-based high-speed microaggitation^14^. Classically, microwave ovens and ultrasound devices have been utilized for rapid IHC^15–17^. However, the quality of immunostaining was reduced, and the specimen was damaged by long-term ultrasonic irradiation^18^.

In this study, we attempted the R-IHC system to facilitate the binding frequency between endogenous Rac/Cdc42-GTP protein and GST-PAK-RBD probe without disturbing conformation of Rac- and Cdc42-GTP. We succeeded in detecting Rac/Cdc42 activation in human colon cancer FFPE tissues with various patterns, strikingly in the invasive front, of which activity was correlated with clinicopathological factors such as lymphatic vessel invasion, raising the value for predicting invasion and metastasis in various types of cancers; although it needs to be noted that the binding to and detection of other Rac members, such as Rac2 and Rac3 and/or Cdc42, cannot be excluded in patient-derived material.

## Methods

### Cells

293T human embryonic kidney cells were cultured in Dulbecco’s modified Eagle’s medium (DMEM, Nissui, Tokyo, Japan) containing 10% fetal bovine serum (FBS, Gibco®-Life Technologies, Grand Island, NY, USA). Two human colon cancer cell lines, HCT116 and WiDr cells, were cultured in McCoy’s 5A (Gibco®-Life Technologies) and RPMI 1640 (Nissui) medium containing 10% FBS, respectively.

### Transfection of Rac1 V12 and Rac1 N17 in 293T cells

For the GST pull-down assay, immunofluorescence, and construction of the cell block, 293T cells were transiently transfected with pCXN2-Flag-Rac1 V12 or pCXN2-Flag-Rac1 N17 using Fugene HD transfection reagent (Promega, Madison, WI, USA).

### GST pull-down assay and immunoprecipitation for detecting activated Rac1

293T cells with or without enforced Rac1 V12 or Rac1 N17 were lysed with lysis buffer (25 mM HEPES (pH 7.4), 150 mM NaCl, 1 mM EDTA, 1% NP40, 10 mM MgCl_2_, 10% glycerol, 1 mM PMSF). For the GST pull-down assay, 1 mg of protein lysate was incubated with 10 µg GST-PAK2 RBD (recombinant protein) with rotation for 30 min at 4 °C, followed by incubation with 20 µl glutathione Sepharose 4B beads (GE Healthcare, Bio-Sciences Uppsala, Sweden) with additional rotation for 30 min at 4 °C. After brief centrifugation, the supernatant was discarded, and the precipitates were washed with lysis buffer three times. Final precipitates were suspended in 20 µl of 2 × SDS sample buffer (100 mM Tris–HCl (pH 6.8), 4% SDS, 10% β-mercaptoethanol, 20% glycerol, and 0.01% BPB), boiled for 5 min, and subjected to electrophoresis using a 12% SDS–PAGE gel. PVDF membrane-transferred separated proteins were immunoblotted with mouse monoclonal anti-Rac1 antibody (Ab) (BD Transduction Laboratories, CA, USA). For immunoprecipitation, 1 mg of protein lysate was incubated with anti-active Rac1 Ab (New East, Malvern, PA, USA) at 4 °C overnight. After brief centrifugation, the subsequent steps were performed as described above.

To reveal activation of Rac1 in human colon cancer cell lines, HCT116 and WiDr cells were serum-starved overnight and stimulated with FBS for 30 min. The cell lysate was subjected to GST pull-down assay as described above.

### Immunofluorescence for activated Rac1 in 293T cells

293T cells expressing Rac1 V12 were cultured on glass-based dishes (IWAKI, Tokyo, Japan) and fixed in 3% paraformaldehyde (Nacalai Tesque, Kyoto, Japan) in PBS for 15 min. The cells were permeabilized with 0.1% Triton X-100 for 4 min and blocked with 1% BSA for 20 min. To detect activated Rac1, the cells were treated with 2 µg/ml GST-PAK-RBD for 2 h at room temperature (RT), followed by anti-GST Ab overnight at 4 °C and Alexa Fluor 488-conjugated anti-mouse IgG (Invitrogen, Carlsbad, CA, USA) for 1 h at RT. In comparison, the fixed and permeabilized cells were stained with anti-active Rac1 Ab (New East) at 4 °C overnight. Images were acquired using a fluorescence microscope (Olympus, Tokyo, Japan).

### Immunostaining of activated Rac1 in 293T cell block

To establish a method for detecting activated Rac1 in formalin-fixed paraffin embedded (FFPE) tissues, optimal conditions of immunostaining were investigated using cell blocks of 293T cells with or without enforced Rac1 V12. 293T cells were transfected as described above and fixed in formalin, and cell blocks were constructed. The concentration of GST-PAK (2, 5, 10 µg/ml), dilution ratio of α-GST Ab (× 50, × 100 dilution), formalin-fixed duration (30 min, 2 h, 1 day, 2 days, 3 days), and requirement of antigen retrieval (pressure cooker: PC, in pH 6.0 citric acid buffer) were investigated. The established protocol was as follows: incubation at 37 °C for 10 min, deparaffinization for 10 min, endogenous peroxidase (3% H_2_O_2_) for 5 min, GST-PAK-RBD for 5 min using an R-IHC machine, 1st Ab (α-GST Ab) at 37 °C for 30 min and for an additional 10 min at RT, polymer 2nd Ab for 30 min (Dako). During each step, specimens were washed with PBS containing 0.5% Tween 20. After coloring with diaminobenzidine (DAB, Dako) within 50 s, the tissues were nuclear stained (Mayer) for 30 s, dehydrated, penetrated, and mounted (Supplementary Fig. 1). In principle, this method also recognizes activated Cdc42.

### Detection of activated Rac and Cdc42 in mouse xenografts of human colon cancer cell lines

HCT116 and WiDr cells (1 × 10^7^) were subcutaneously injected into six- to eight-week-old female 10 nude mice, Balb/cA Jcl nu/nu (Clea Japan, Inc., Tokyo, Japan) per cell type. After approximately one month, the mice were sacrificed by cervical fracture under isoflurane inhalation anesthesia, and the growing tumor tissues were resected. The tumors that grew to almost equal volume were randomly divided into two groups within the weight range group (3 mice/group) and fixed in formalin or the PAXgene Tissue system (Qiagen), which is a formalin-free system designed to improve the quality of molecular analysis without disturbing those of histopathological analysis. Rac and Cdc42 activation in the excised tumors was evaluated as described above. All animal experiments were conducted in accordance with the guidelines of the Hokkaido University Manual for Implementing Animal Experimentation, which was consistent with the ARRIVE Guidelines for the Care and Use of Laboratory Animals and for study design and approved by the Institutional Animal Care and Use Committee at Hokkaido University Graduate School of Medicine (Number 12-0092). All researchers who performed procedures using live animals were preapproved by the Animal Welfare Committee of Hokkaido University. For each animal experiment, four different investigators were involved as follows: two investigators (RH, LW) performed the surgical procedure and all steps for making tumor specimens and were aware of treatment group allocation (formalin or PAX gene fixation). Two investigators (MaTs, MiTa) who were unaware of treatment evaluated the staining intensity and pattern of active Rac1/Cdc42.

### Analysis of activated Rac and Cdc42 in human colon cancer tissue microarray (TMA) specimens

In tissue microarray (TMA) specimens from 33 patients with colon cancer (Stage 0–IV: 33 cases) diagnosed at the Department of Cancer Pathology, Hokkaido University Graduate School of Medicine between 2003 and 2015 with informed consent from participants, activated Rac and Cdc42 were immunostained, as shown in Supplementary Fig. 1. DAB coloring indicating Rac/Cdc42 activity was analyzed in the tumor area and normal mucosa using Histoquest software (Novel Science Co., Ltd, Tokyo, Japan). Correlations between Rac/Cdc42 activity and lymphatic vessel invasion, venous invasion, and lymph node metastasis were analyzed. Human studies followed the ethical guidelines for clinical application, in accordance with the Declaration of Helsinki. All ethical issues related to human pathological specimens were discussed and approved by the Ethics Committee of Hokkaido University Graduate School of Medicine (Number 15-022).

### Analysis of activated Rac and Cdc42 in the human colon cancer FFPE tissues

The colon cancer FFPE tissues of 50 patients diagnosed with colon cancer at the Department of Cancer Pathology, Hokkaido University Graduate School of Medicine between 2003 and 2015 with informed consent from participants are summarized in Supplementary Table 1 (Stage 0: 3 cases; Stage I, II, IIIA, and IV: each 10 cases; and Stage IIIB: 7 cases). Activated Rac and Cdc42 were immunostained and evaluated with the R-IHC system as described above. In identical patients, Rac/Cdc42 activity in normal mucosa and tumor areas was measured, and the activation patterns of Rac/Cdc42 were investigated in the tumor areas. Statistical analyses were performed between the Tis, T1, and T2 groups (low invasion) and the T3 and T4 groups (high invasion) using Student’s t test. A value of *P* < 0.05 was considered statistically significant. All ethical issues related to human pathological specimens were discussed and approved by the Ethics Committee of Hokkaido University Graduate School of Medicine (Number 15-022).

### Statistical analysis

Graphical data are presented as the mean and standard deviation (S.D.), and Student’s t test was used for comparisons, with *P* < 0.05 considered significant. The F test was used to investigate the equal or unequal variance of samples.

## Results

### Higher sensitivity and specificity of active Rac and Cdc42 detection using GST-PAK in immunofluorescence analyses

**Figure 1:**
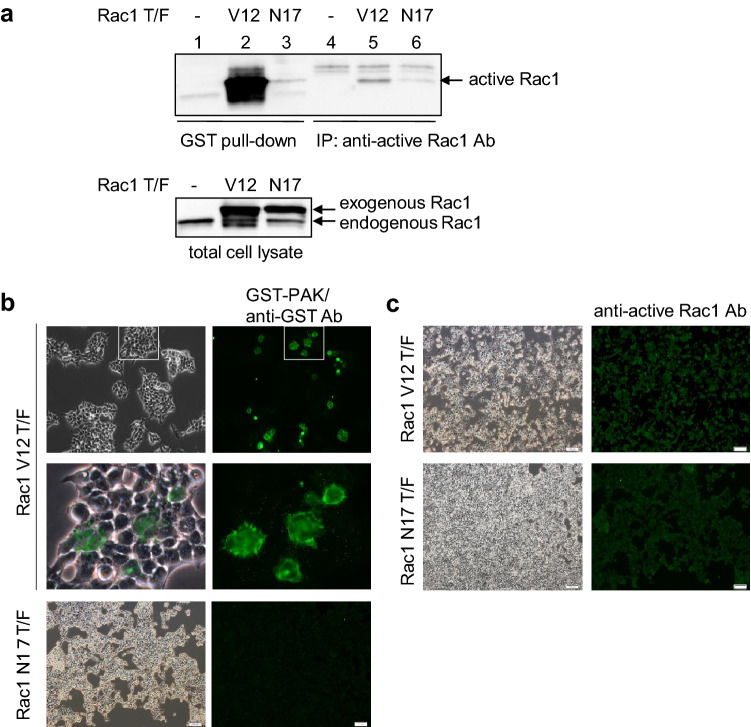
Comparison between the GST-PAK-RBD probe and anti-active Rac1 antibody for active Rac1 detection. 293T cells were transiently transfected with or without expression vectors of pCXN2-Flag-Rac1V12 or pCXN2-Flag-Rac1N17. (**a**) GTP-bound activated Rac1 (Rac1-GTP) was detected by GST pull-down assay using GST-PAK-RBD (Lanes 1–3) or immunoprecipitation using anti-active Rac1 antibody (Ab) (New East, Lanes 4–6), followed by immunoblotting (IB) of Rac1. The expression levels of total Rac1 were examined using an anti-Rac1 Ab (lower). (**b**,**c**) 293T cells with enforced Rac1V12 or Rac1N17 were subjected to immunofluorescence analysis using GST-PAK-RBD/anti-GST Ab (**b**) and anti-active Rac1 Ab (**c**). In (**b**), high magnification images of the areas surrounded by squares are shown lower. Green fluorescence image representing Rac1V12-expressing cells (right) and merged image to phase-contrast micrograph (left) are displayed.

In the process of tumor progression, the detection of active Rac/Cdc42 is useful for a better understanding of the migration and invasion abilities of tumor cells and the prediction of metastasis and patient prognosis. A GST pull-down assay using GST-PAK-RBD is available for the biochemical detection of active Rac and Cdc42. Recently, an anti-active Rac1 antibody (Ab) has been made commercially available. Here, we compared the detection sensitivity and specificity of active Rac1 between a GST pull-down assay and immunoprecipitation using an anti-active Rac1 Ab. In 293T cells, exogenous Rac1 V12, but not N17, was effectively detected in the GST pull-down assay, whereas immunoprecipitation using an anti-active Rac1 Ab failed (Fig. 1a).

By immunofluorescence analysis, the combination of GST-PAK-RBD and anti-GST Ab clearly visualized active Rac1 in 293T cells transfected with Rac1 V12, showing developed membrane ruffling and lamellipodia (Fig. 1b). Meanwhile, anti-active Rac1 Ab exhibited nonspecific staining in Rac1 V12- and N17-expressing cells (Fig. 1c). These results indicate an advantage of GST-PAK-RBD as a detection probe for active Rac1, in principle also for active Cdc42, in terms of both specificity and sensitivity.

### Establishment of R-IHC-based detection of active Rac and Cdc42 on FFPE specimens

**Figure 2:**
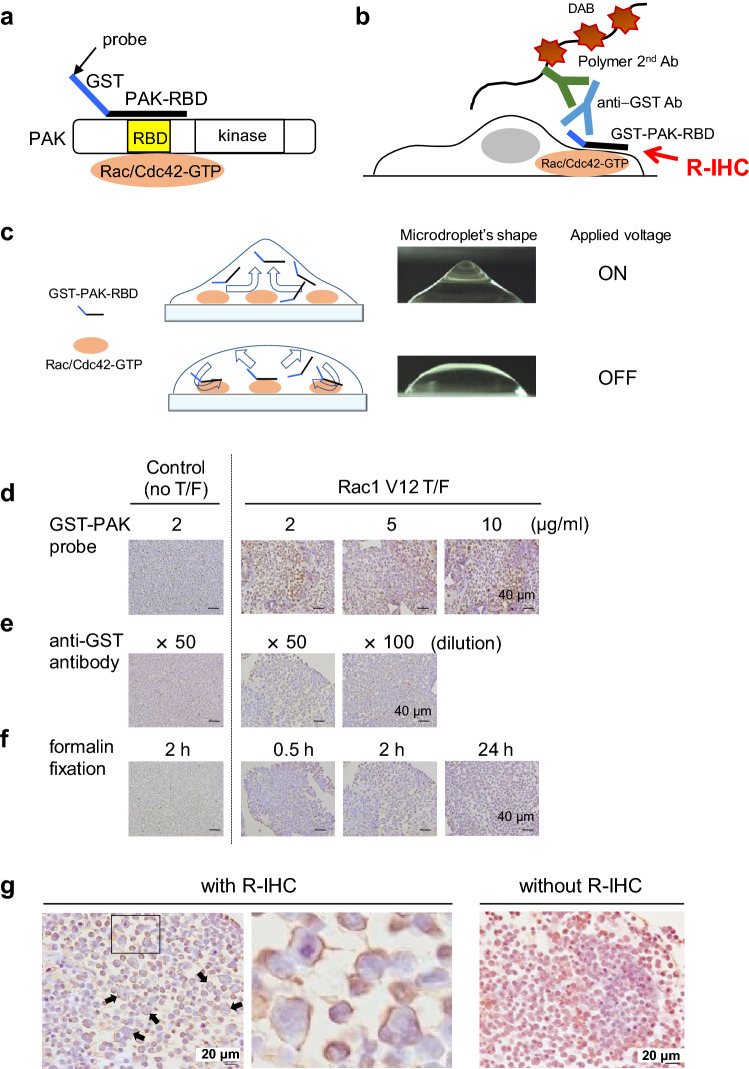
Staining of active Rac1/Cdc42 using a combination of GST-PAK RBD probe and R-IHC machine. (**a**) Structures of PAK protein and GST-PAK-RBD probe are displayed. (**b**) Principle of immunostaining for active Rac and Cdc42 using the GST-PAK-RBD probe on FFPE tissues. An R-IHC machine was employed to promote the reaction between Rac/Cdc42-GTP and the GST-PAK-RBD probe. (**c**) Schematic of the changes in the microdroplet as the voltage is switched on and off. The GST-PAK-RBD probes are mixed within the microdroplet. The resultant Coulomb force stirs the probe solution on the sections, and the opportunity for binding between the probe and Rac/Cdc42-GTP is increased because as the voltage is turned on and off at regular intervals, the microdroplet shape is changed. (**d**–**g**) Establishment of optimal staining conditions for active Rac1, rationally active Cdc42 too, in FFPE samples. A cell block from 293 T cells with Rac1V12 was utilized for the following investigations: (**d**) GST-PAK concentration (2, 5, 10 µg/ml), (**e**) dilution ratio of α-GST Ab (× 50, × 100 dilution), (**f**) formalin-fixed duration (30 min, 2 h, 1 day), and (**g**) immunostaining with or without R-IHC. In (**g**), high magnification image is shown in middle. Arrows indicate cells with enforced Rac1V12. 293T cells without transfection were used as a negative control.

Although GST-PAK-RBD acts as an effective probe for detecting active Rac1, rationally also active Cdc42 biochemically and immunofluorescence in living cells (Fig. 1), this probe could not serve positive staining results on sections for histopathological evaluation. To establish a new technique visualizing Rac/Cdc42 activation in FFPE cancer tissues, various conditions, such as the concentration of the GST-PAK probe, dilution ratio of anti-GST Ab, and duration of formarin fixation, were optimized using 293T FFPE cell blocks with enforced Rac1 V12. Here, we employed an R-IHC machine to enhance the binding frequency between Rac/Cdc42-GTP and GST-PAK-RBD (Fig. 2a–c). In this system, the GST-PAK-RBD probes are mixed within the microdroplet, and the voltage is switched on and off. The resultant Coulomb force stirs the probe solution on the sections, and the opportunity for binding between the probe and Rac/Cdc42-GTP is increased because as the voltage is turned on and off at regular intervals, and the microdroplet shape is changed (Fig. 2c). Excellent staining of active Rac1 was obtained under the following conditions: 2 µg/ml GST-PAK-RBD (Fig. 2d), 50-fold dilutions of anti-GST Ab (Fig. 2e), and 24 h of formalin fixation (Fig. 2f). Under these conditions, the specific membrane activation of Rac1, also including endogenous active Cdc42, was clearly detected with R-IHC (Fig. 2g, left and middle). Without the use of the R-IHC machine, only nonspecific staining was observed (Fig. 2g, right). Meanwhile, no signal was detected in FFPE cell blocks of 293T cells enforced with Rac1 N17 (Supplemental Fig. S2). Thus, the R-IHC system seems to be essential for detecting active Rac and reasonably Cdc42 in FFPE tissues. Of note, active Rac/Cdc42 staining was completely lost by antigen retrieval using a pressure cooker (data not shown). In addition, GST-RalGDS-RBD and GST-Rhotekin-RBD probes targeting active Rap1 and active Rho, respectively, did not react with active Rac1 (Supplementary Fig. S3), suggesting the high specificity of the GST-PAK-RBD probe. The eventually established protocol is shown in Supplemental Fig. 1. Notably, the enforced Cdc42 V12, but not active Rac2, appears to be able to bind to the GST-PAK probe (Supplementary Fig. S4). On the other hand, under FBS stimulation in human colon cancer cell lines, the activity of endogenous Cdc42 was much lower than that of Rac1 (Supplementary Fig. S5a), and the Rac1 inhibitor NSC23766 clearly reduced membrane staining in FFPE cell blocks with the GST-PAK probe (Supplementary Fig. S5b), suggesting that this system can in principle detect both Rac and Cdc42, but here is likely to reflect the activation of endogenous Rac1.

### Visualization of active Rac and Cdc42 in xenograft tumors of human colon cancer cell lines

**Figure 3:**
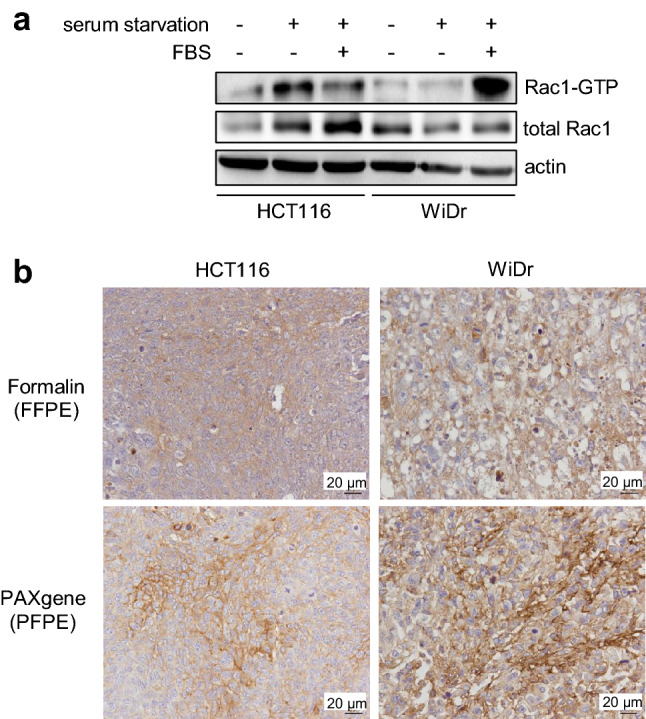
(**a**) Expression and activation levels of Rac and Cdc42 in the human colon cancer cell lines HCT116 and WiDr. The cells were treated as follows: Lanes 1 and 4: without serum starvation and FBS stimulation; Lanes 2 and 5: only serum starvation overnight; Lanes 3 and 6: serum starvation overnight and the subsequent FBS stimulation for 30 min, and GST pull-down assay was performed. (**b**) HCT116 and WiDr cells were subcutaneously implanted in nude mice, formed tumors were fixed in formalin (upper, FFPE) or PAXgene (lower, PFPE), and immunostaining for active Rac and Cdc42 was performed with an R-IHC system.

We next examined the intensity and staining pattern of active Rac/Cdc42 staining in mouse xenograft tumors derived from human colon cancer cell lines. GST pull-down assays demonstrated that HCT116 and WiDr cells had increased activation of Rac1 with or without FBS stimulation (Fig. 3a), and these cells were implanted subcutaneously in nude mice. Activated Rac/Cdc42 was detected in formed tumor FFPE tissues by the R-IHC-based staining (Fig. 3b, upper panels). Notably, PAXgene fixation solution (QIAGEN) is likely to provide a great advantage for membrane staining of activated Rac/Cdc42 (Fig. 3b, lower panels).

### Rac and Cdc42 are activated with various patterns in human colon cancer FFPE tissues and correlates with tumor invasion

**Figure 4:**
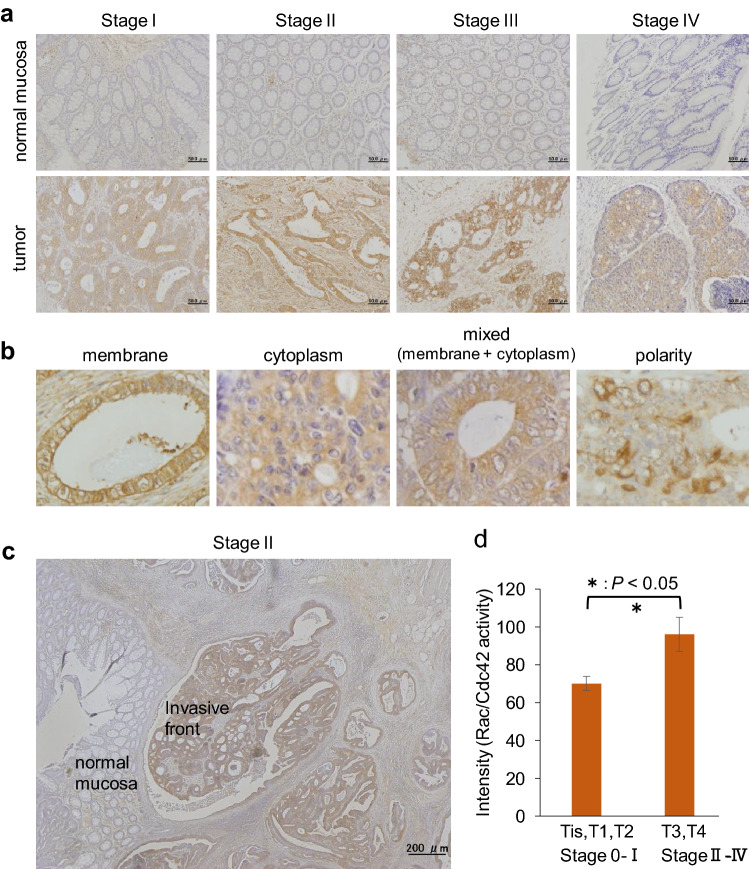
Rac1 activity in human colon cancer FFPE tissues correlates with tumor invasion. Immunostaining for active Rac and Cdc42 was performed in 50 human colon cancer FFPE tissues with the R-IHC system. (**a**) In identical patients, Rac/Cdc42 activity in normal mucosa (upper) and tumor area (lower) are displayed. (**b**) Micrographs and illustrations of representative activation patterns of Rac/Cdc42 are shown, designated as membrane, cytoplasm, mixed type of membrane and cytoplasm, and polarized distributions. Brown (DAB) and blue (hematoxylin) colors indicate Rac/Cdc42 activity and the nucleus, respectively. (**c**) Rac/Cdc42 activity in the invasive front of the tumor is shown. (**d**) DAB intensities showing Rac/Cdc42 activity were measured using Histoquest software, and the average values in two groups divided into Tis, T1, T2 and T3, T4 are shown with standard deviation.

Under optimal staining conditions (Fig. 2), we succeeded in detecting activated Rac and Cdc42 in colon cancer FFPE tissue sections and breast and brain cancers (Supplemental Fig. S6). We examined Rac/Cdc42 activity in 50 human colon cancer FFPE tissues (Stage 0–IV, Supplemental Table S1). Activated Rac/Cdc42 was detected in tumor cells in all stages (Fig. 4a) and was especially higher in stage IV (Supplemental Fig. S7), whereas no activity was found in the corresponding normal mucosa (Fig. 4a). Notably, various activation patterns of Rac/Cdc42 were observed in colon cancer FFPE tissues, designated plasma membrane, cytoplasmic, mixed membrane and cytoplasm, and polarized patterns (Fig. 4b, Supplemental Table S2), suggesting functional variation of activated Rac and Cdc42 in human colon cancer tissues. In addition, Rac/Cdc42 activity was higher in the invasive front of the tumor than in the central region (Fig. 4c, Supplemental Fig. S8). Rac/Cdc42 activity seems to be correlated with tumor invasion status evaluated by TNM classification. Rac/Cdc42 activity was significantly higher in the T3 and T4 groups than in the Tis, T1, and T2 groups (Fig. 4d).

### Activation of Rac and Cdc42 in human colon cancer with lymphatic vessel and venous invasion and lymph node metastasis

**Figure 5:**
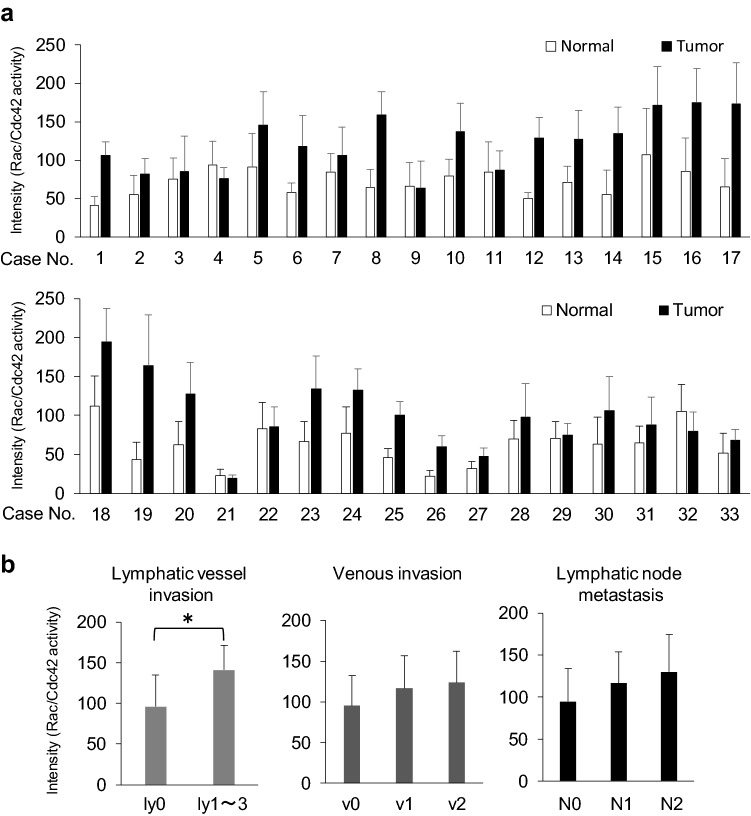
Immunostaining for active Rac and Cdc42 was performed in tissue microarray (TMA) samples from 33 patients with colon cancer. (**a**) DAB intensities showing Rac/Cdc42 activity in the tumor area and normal mucosa were measured using Histoquest software and graphed as the means ± SD. (**b**) Correlations between Rac/Cdc42 activity and lymphatic vessel invasion (left), venous invasion (middle), and lymph node metastasis (right) were analyzed and graphed as the means ± SD. *P < 0.05.

We next investigated the association between Rac/Cdc42 activity and clinicopathological factors in colon cancer. For this analysis, tissue microarray (TMA) samples from 33 patients with colon cancer were utilized. In 29 cases, Rac/Cdc42 activity was increased in the tumor region compared to normal mucosa (Fig. 5a, Supplemental Fig. S9, average Rac/Cdc42 activity, normal mucosa *versus* tumor = 1: 1.86). Meanwhile, in the remaining 4 cases (Case No. 4, 9, 21, 32), Rac/Cdc42 activity was reversed (normal mucosa *versus* tumor area = 1: 0.85). Actual intensities of active Rac/Cdc42 in normal mucosa and tumor were as follows: 67.29 and 110.92 in all cases, 66.64 and 117.97 in 29 cases (normal mucosa < tumor), 72.01 and 59.83 in 4 cases (normal mucosa > tumor). Of note, positive correlations between Rac/Cdc42 activity and lymphatic vessel invasion, venous invasion, and lymph node metastasis were detected (Fig. 5b).

## Discussion

Rac and Cdc42 are signaling proteins regulating the actin cytoskeleton. Activated Rac and Cdc42 bind to the PAK effector protein and transmit signaling and promote motility, invasion, and metastasis of cancer cells. The splice variant of Rac1 has been reported to be highly expressed in breast and colon cancer^9^.

In this study, we succeeded in developing a novel method to visualize activated Rac and Cdc42 in human cancer FFPE tissues using the R-IHC technique. The deciding factor of success is owed to the accelerated binding between endogenous Rac/Cdc42-GTP protein and GST-PAK probe without disrupting the conformation of the GTP-bound proteins by marked microaggitation in the electric fields, leading to enhanced Brownian motion^19,20^. In conventional immunohistochemical staining, antigen retrieval is often performed by heating the treatment at high pressure; however, this probably causes the dissociation of GTP from GTP-bound forms. Meanwhile, the background staining when R-IHC was not applied is suggested to be due to the nonspecific deposition of a substantial amount of GST-PAK on the tissues. Thus, the facilitation of binding frequency between Rac/Cdc42-GTP and GST-PAK using R-IHC technique in the short term seems to be essential for the successful detection of activated Rac/Cdc42 in FFPE tissues. Notably, the R-IHC system with the GST-PAK probe was capable of detecting both exogenously expressed active Rac1 and active Cdc42 (Supplementary Fig. S4b), which is reasonable because it can bind to both active forms of Rac and Cdc42 in principle based on the homology of the binding site. Biochemical approach in combination using antibodies against Rac and Cdc42 may complement to distinguish between activated Rac and Cdc42 partially. However, it should be noted that anti-Rac1 antibody commercially available sometimes crosses Cdc42 too due to the high homology of the antigen recognition site (Supplementary Fig. S4a). Therefore, at present, this assay cannot be used to conclusively determine Rac and/or Cdc42 activation. On the other hand, in the human colon cancer cells we tested, the endogenous activity of Cdc42 appeared to be much lower than that of Rac1 under physiological conditions (Supplementary Fig. S5). Thus, this system might be likely to reflect the activation of endogenous Rac1 mainly in the patients’ tumor tissues, although activation of other Rac members, such as Rac2 and Rac3 and/or Cdc42 cannot be ruled out.

The most direct method to measure the activities of small GTPases is P*i*-orthophosphate labeling, followed by immunoprecipitation of the GTPases and thin-layer chromatography for the separation of GDP and GTP^21^. Application of this method is limited because of rapid GTP hydrolysis, a lack of efficient antibodies to precipitate the GTPases, and most critically the use of hazardous isotopes. The most popular method is the so-called pull-down assay, in which GTP-bound small GTPases are precipitated by the GST-fused effector molecules^22^. The GST pull-down assay ensures detection with high sensitivity and specificity, however, it completely abolishes the spatial information due to cell lysis. To resolve this issue, a method using intramolecular fluorescence resonance energy transfer (FRET) biosensors has been developed^23,24^. FRET technology enables the visualization of several intracellular events in living cells; Kiyokawa et al. demonstrated that Rac1 suppression at the apical membrane is essential for cyst structure maintenance using a FRET-based live imaging system^25^. Meanwhile, proficient skills to develop biosensors, uptake of FRET probes into living cells, and expensive equipment to detect FRET reactions are required. The R-IHC technique using a GST-PAK probe easily and rapidly enables the detection of activated Rac and Cdc42 in FFPE tissues, which contributes to realizing special activation of Rac/Cdc42 in cancer tissues. This technique can be applied to other small GTPases, such as Rho and Rap1, in addition to the Ras family, by proper combination with probes. Meanwhile, it should be noted that Baker and colleagues have conclusively shown that the active Rac1 antibody (used in this manuscript too) does not detect active Rac1 at all but seems to bind to vimentin^26^. This is supportive information for our results (Fig. 1c) and is important to help avoid reaching inaccurate conclusions. Recently, the EGFR-targeting monoclonal antibody cetuximab has been administered to colon cancer patients without *KRAS* mutations. Although it takes several days for sequencing-based mutation analysis, our established method with R-IHC enables us to reveal Ras activation within 30 min by the use of a GST-Raf probe (data not shown), leading to rapid application of cetuximab for personalized medicine. In addition, rapid immunohistochemistry (IHC) for Ki-67/MIB-1, CK (AE1/AE3), CK7, CK20, EMA, TTF-1, CD10, GFAP, CD20, and CD30 is enabled within 20 min using this device, which provides valuable information for intraoperative rapid pathological diagnosis^27–29^. Recently, the usefulness of this device has been reported in the fields of rapid dual in situ hybridization (RISH) targeting HER2, scoring of programmed death ligand 1 (PD-L1), and cell biology^30–33^. In the future, our newly developed method raises the possibility of providing valuable information in many fields, not only in cancer but also embryology, cell biology, pathobiology of various diseases, pathological diagnosis, and so on. We also found special activation of Rac and Cdc42 in the fundic gland (Supplemental Fig. S10), although the significance has remained unknown.

Of note, in TMA samples from 33 patients, we demonstrated positive correlations between Rac/Cdc42 activity of cancer cells and clinicopathological factors such as lymphatic vessel invasion (Fig. 5b). Rac/Cdc42 activity also seems to be positively associated with venous invasion and lymph node metastasis (Fig. 5b), although they were not statistically significant due to a likely insufficient number of samples to analyze. In addition, in 50 colon cancer FFPE tissues, Rac/Cdc42 was further activated in the invasion front of the tumor (Fig. 4c). Recently, it was reported that patients with RacGAP1 (Rac GTPase activating protein 1) expression in the invasive front of the tumor exhibited a significantly poorer prognosis than those without RacGAP1 expression^34^. Thus, the detection of activated Rac1 in cancer tissues enables the prediction of the invasive property of tumor cells, forthcoming metastasis, and patient prognosis, thereby making it a useful biomarker.

In this study, we newly found various activation patterns of Rac and Cdc42 in colon cancer FFPE tissues, designated as membrane, cytoplasm, mixed type of membrane and cytoplasm, and polarized patterns (Fig. 4b, Supplemental Table S2), which may demonstrate distinct functions of activated Rac and Cdc42. Predictively, the membrane pattern and polalized distribution might reflect collective invasion and focal activation of tumor cells, respectively. In those cases, more careful follow-up will be needed. The pathobiological significance of these variations should be clarified in the future. NSC23766, a Rac1 inhibitor that prevents the functions of Rac1 GEFs such as Tiam and Trio, is available in an in vitro experimental setting (Supplementary Fig. S5); thus, the detection of activated Rac1 in tumor tissues would be linked to an option of molecular therapy targeting activated Rac1.

In conclusion, this is the first study to detect activated Rac and Cdc42 in human cancer FFPE tissues using the R-IHC system. Rac/Cdc42 activity was significantly higher in the tumor area than in the normal mucosa in colon cancer patients, especially in the invasive front, increasing its usefulness in the prediction of invasion and metastasis in various types of cancers. We expected that this technology would also provide valuable information for other small GTPases, such as Ras, which collectively leads to effective personalized medicine.

## Supplementary Information

Supplementary Information.
