# Supplementary material for: Novel rapid immunohistochemistry using an alternating current electric field identifies Rac and Cdc42 activation in human colon cancer FFPE tissues
Source: Sci Rep. 2022 Feb 2;12:1733. doi: 10.1038/s41598-022-05892-7 (PMC8810803; doi:10.1038/s41598-022-05892-7)
Supplement: Supplementary file 1 — Supplementary Information. [file 41598_2022_5892_MOESM1_ESM.pdf]

Supplementary Figure 1

fixation
↓
deparaffinization
↓ PBS
endogenous peroxidase (RT, 5 min)
↓ PBS
R-IHC → GST-PAK-RBD (RT, 5 min)
↓ PBS
1st Ab (anti-GST Ab, 37℃, 30 min → RT, 10 min)
↓ PBS
polymer 2nd Ab (RT, 30 min)
↓ PBS
DAB coloring
↓ wash
nuclear staining (Mayer)
↓ wash, 10 min
dehydration・penetraion
↓
mounting

Supplementary Figure 1

Optimized protocol of immunostaining for activated Rac/Cdc42 is shown. R-IHC machine is employed to promote reaction between Rac/Cdc42-GTP and GST-PAK-RBD probe. Activated Rac/Cdc42 shows activation of both Rac members and Cdc42.

Supplementary Figure 2

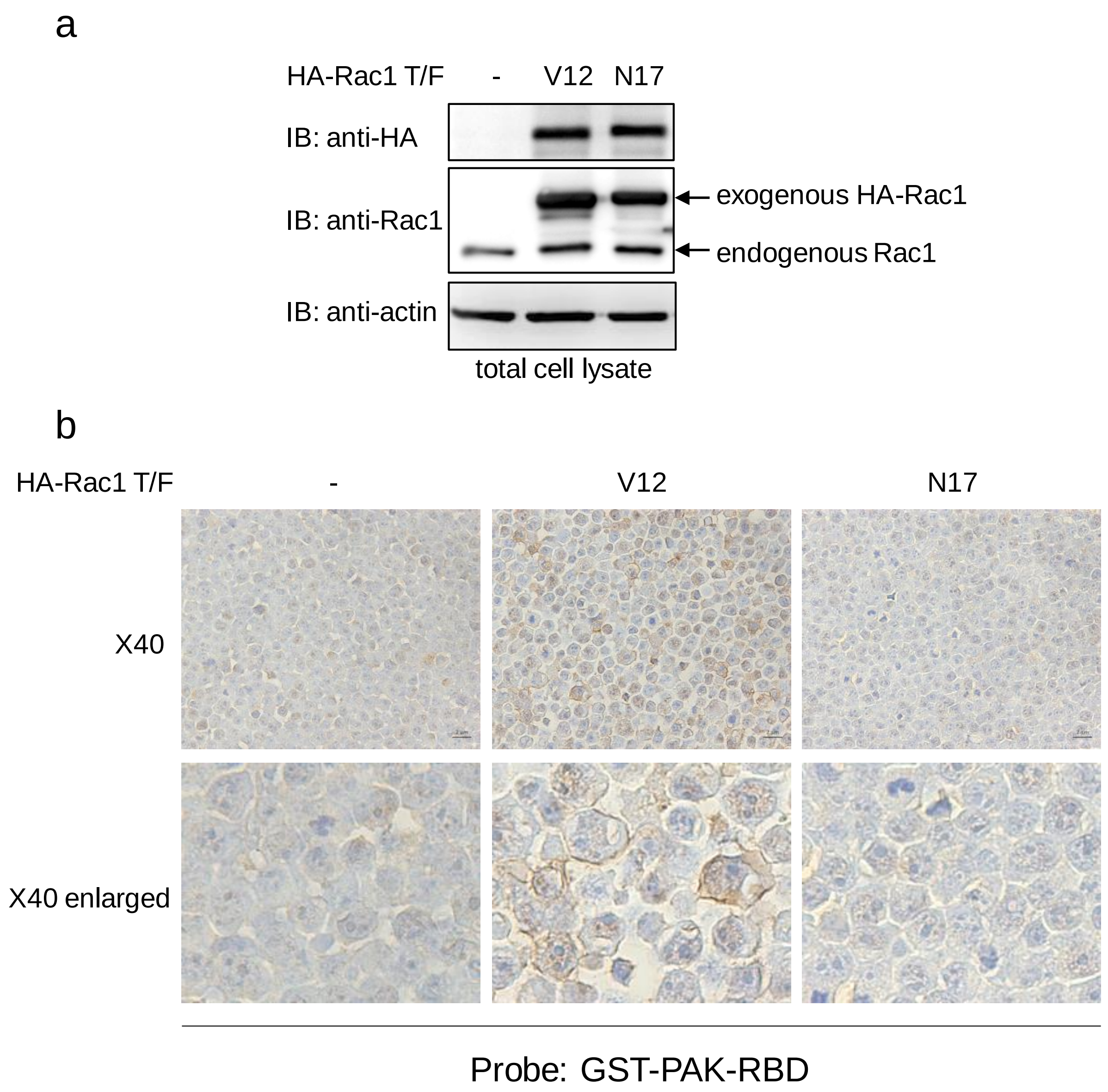

Supplementary Figure 2

a, 293T cells were transfected with or without pEB-HA-Rac1 V12 or N17, and Western blotting demonstrated that almost equal amount of exogenous Rac1 was expressed.

b, After constructing the FFPE cell blocks, R-IHC with GST-PAK-RBD probe was performed under the optimal conditions for detecting active Rac/Cdc42 determined in Figure 2. 293T cells without transfection were used as a negative control. Active Rac/Cdc42 shows activation of both Rac members and Cdc42.

Supplementary Figure 3

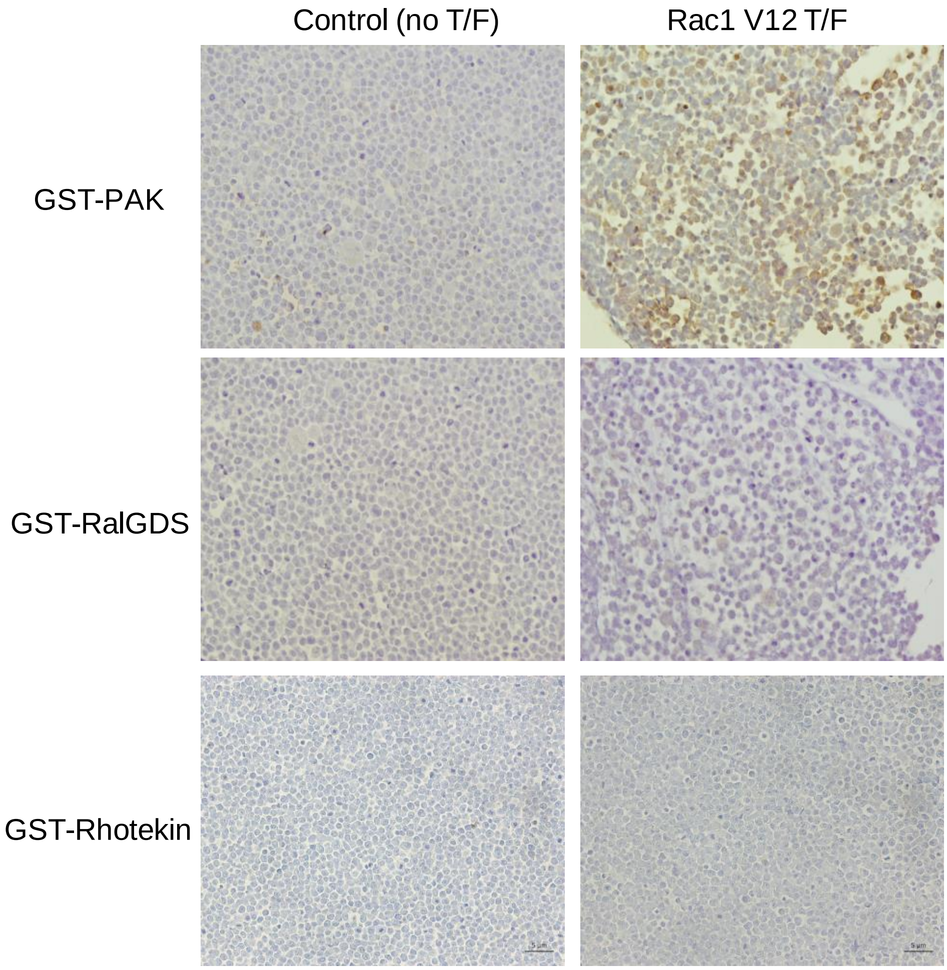

Supplementary Figure 3

Cell block from 293T with Rac1V12 was treated with GST-PAK, GST-RalGDS, and GST-Rhotekin probes, and the subsequent staining was performed. 293T cells without transfection were used as a negative control.

Supplementary Figure 4

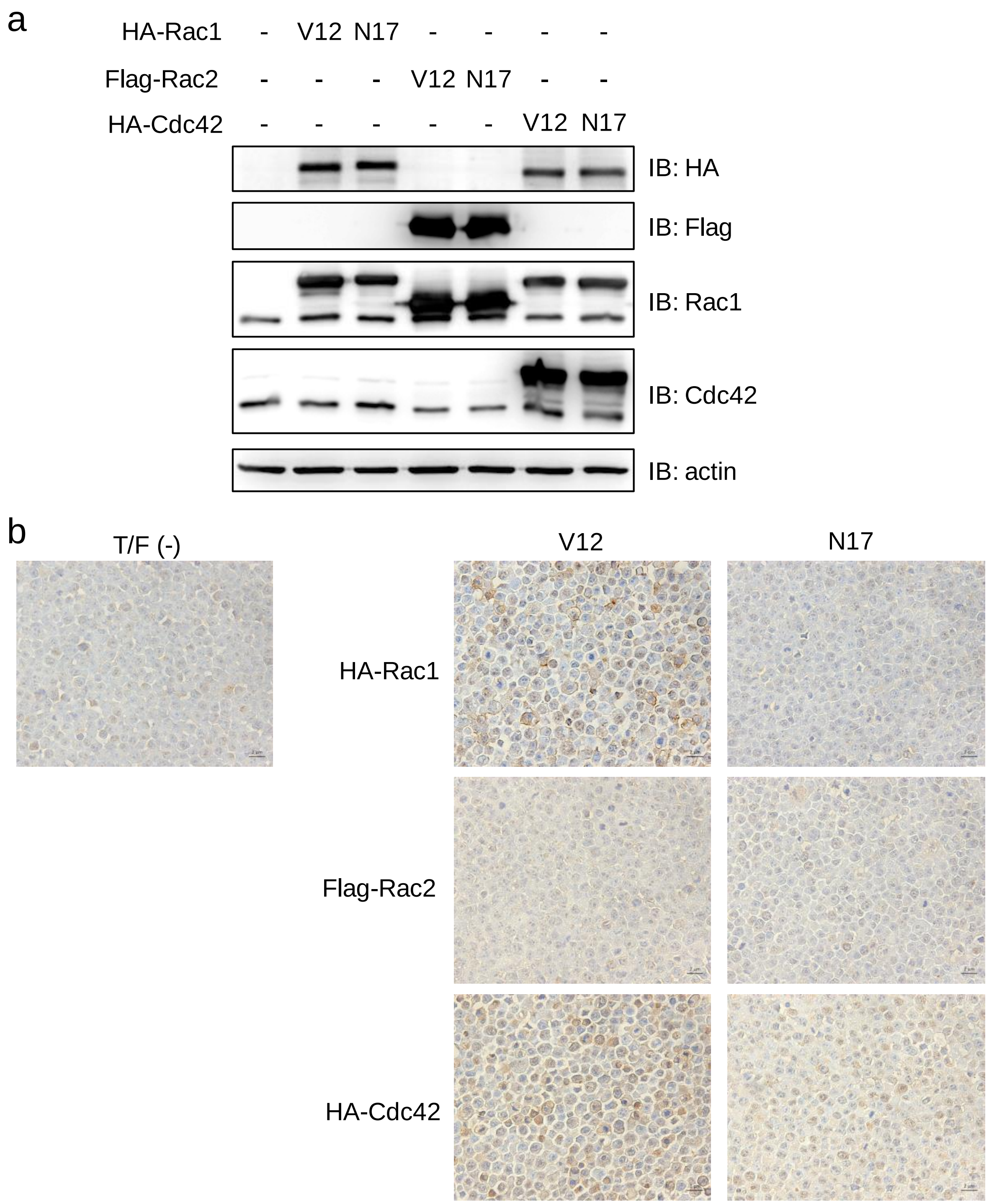

Supplementary Figure 4

a, 293T cells were transfected with pEB-HA-Rac1 V12 or N17, pCXN2-Flag-Rac2 V12 or N17, and pEB-HA-Cdc42 V12 or N17. Western blotting demonstrated that almost equal amount of exogenous proteins were expressed. Anti-Rac1 antibody cross-reacted to the enforced Rac2 and Cdc42 in addition to Rac1 due to high alignment of the immunogen to Rac2 and Cdc42 proteins.

b, After constructing the FFPE cell blocks, R-IHC with GST-PAK-RBD was performed under the optimal conditions shown in Figure 2. 293T cells without transfection were used as a negative control.

Supplementary Figure 5

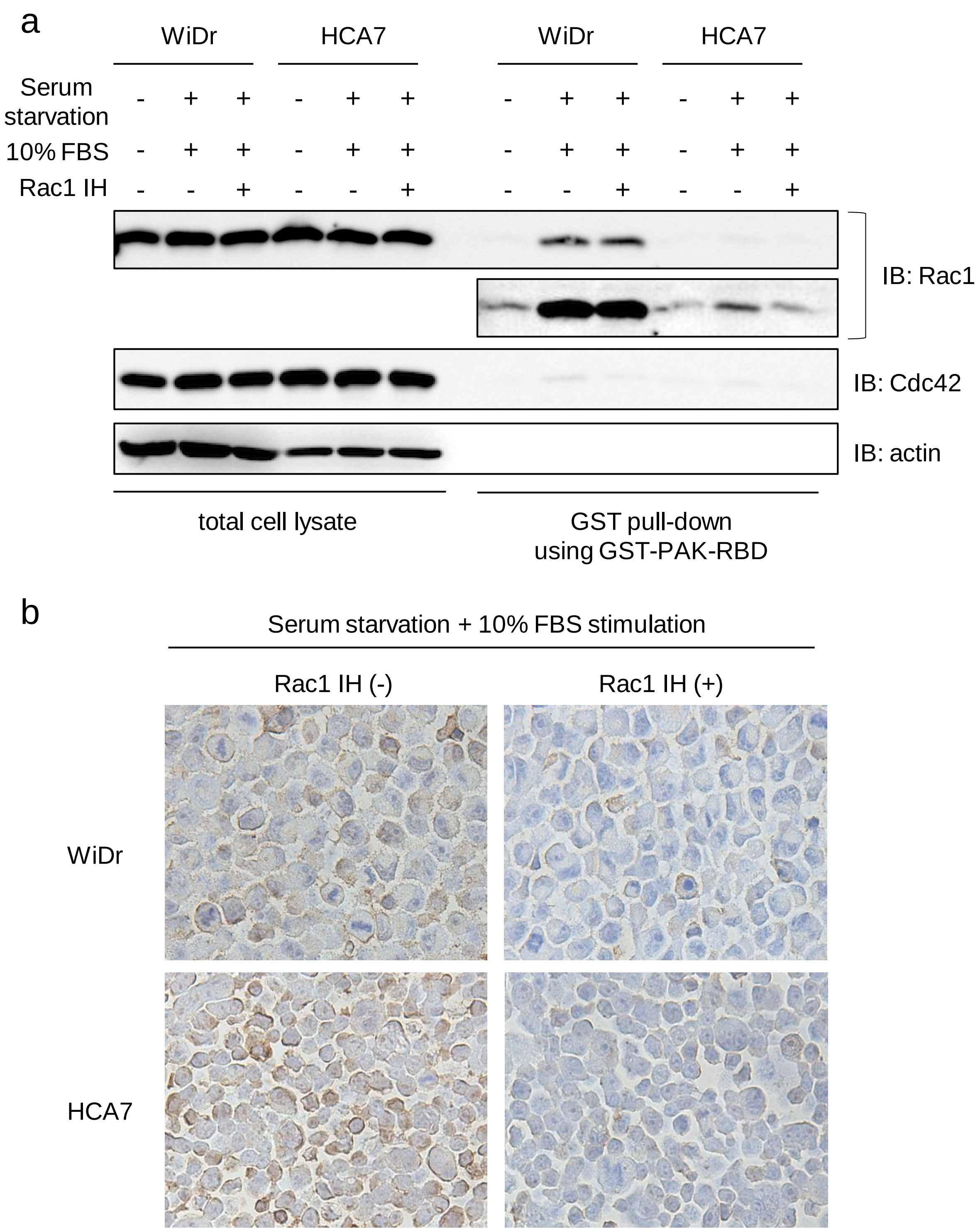

Supplementary Figure 5

a, Colon cancer cell lines WiDr and HCA7 were serum-starved overnight and stimulated with 10% FBS in the presence or absence of 50 μM NSC23766 for 30 min. GST-pull down assay using GST-PAK-RBD and the following Western blotting were performed.

b, Cell block from WiDr and HCA7 with or without NSC23766 Rac1 IH was treated with GST-PAK-RBD probe, and the subsequent staining was performed.

Supplementary Figure 6

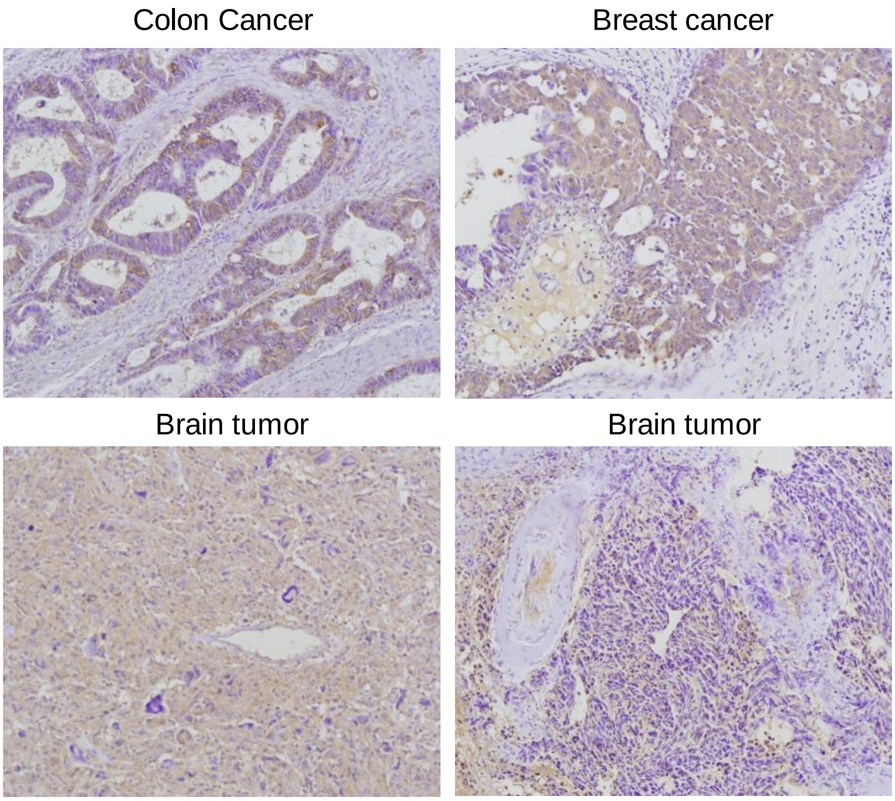

Supplementary Figure 6
Active Rac/Cdc42 on human colon cancer, breast cancer, and brain tumor FFPE tissues was immunostained by optimized protocol as shown in Supplementary Figure 1. Active Rac/Cdc42 shows activation of both Rac members and Cdc42.

Supplementary Figure 7

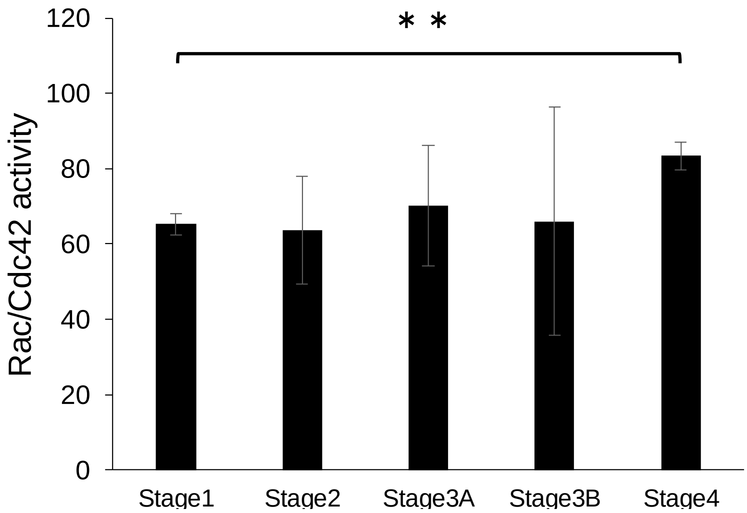

Supplementary Figure 7

In 50 cases of human colon cancer FFPE tissues (Stage 1 – Stage 4), DAB intensities showing Rac/Cdc42 activity in each stage were measured using Histoquest software and graphed as means ± SD. $^{**}P < 0.01$. Rac/Cdc42 activity shows activation of both Rac members and Cdc42.

Supplementary Figure 8

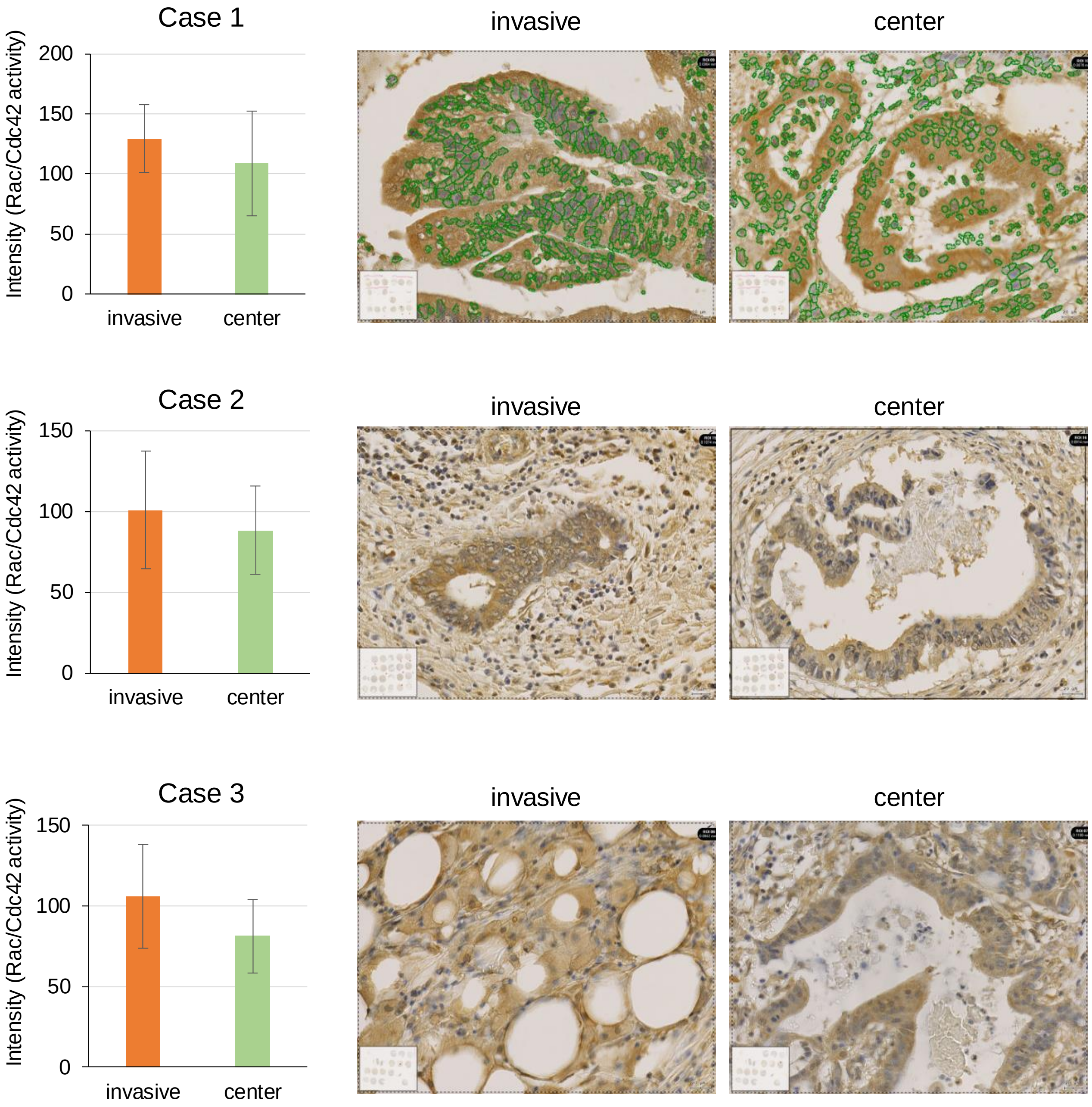

Supplementary Figure 8

On human colon cancer FFPE tissues, Rac/Cdc42 activity was compared between invasive front of tumor and central regions, and graphed as means ± SD (left). Representative photomicrographs of active Rac/Cdc42 staining are shown in 3 cases (right). Rac/Cdc42 activity shows activation of both Rac members and Cdc42..

Supplementary Figure 9

Case 19: Normal＜Tumor

Normal | Tumor

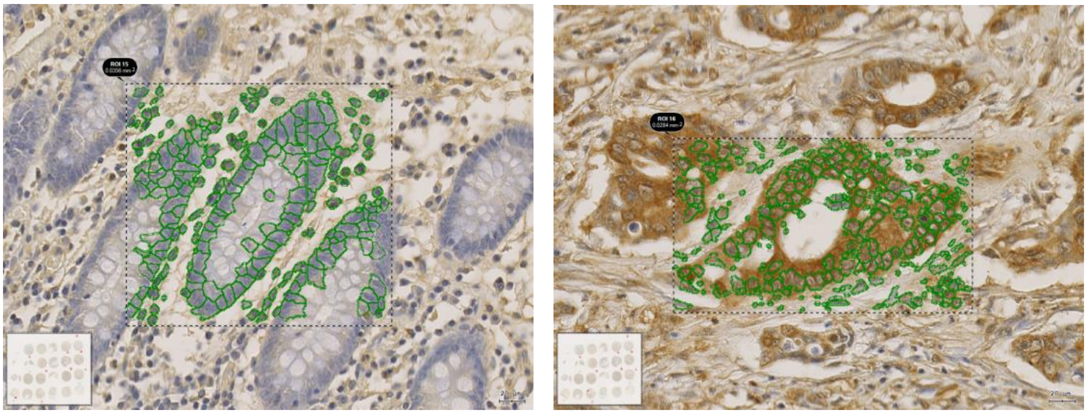

Case 32: Normal＞Tumor

Normal | Tumor

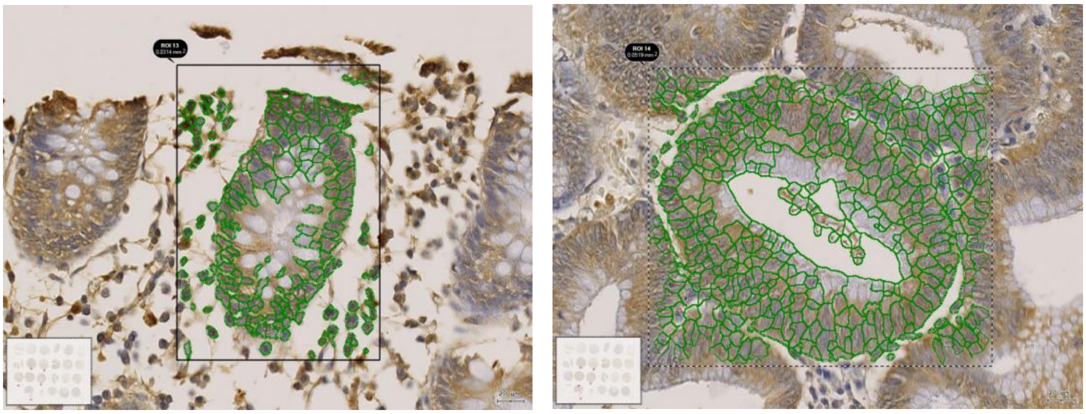

Supplementary Figure 9

Immunostaining for active Rac/Cdc42 were performed in TMA samples from 33 patients with colon cancer. In case 19 and case 32 shown in Fig 5A, the representative photomicrographs in tumor area and normal mucosa are displayed. Active Rac/Cdc42 shows activation of both Rac members and Cdc42.

Supplementary Figure 10

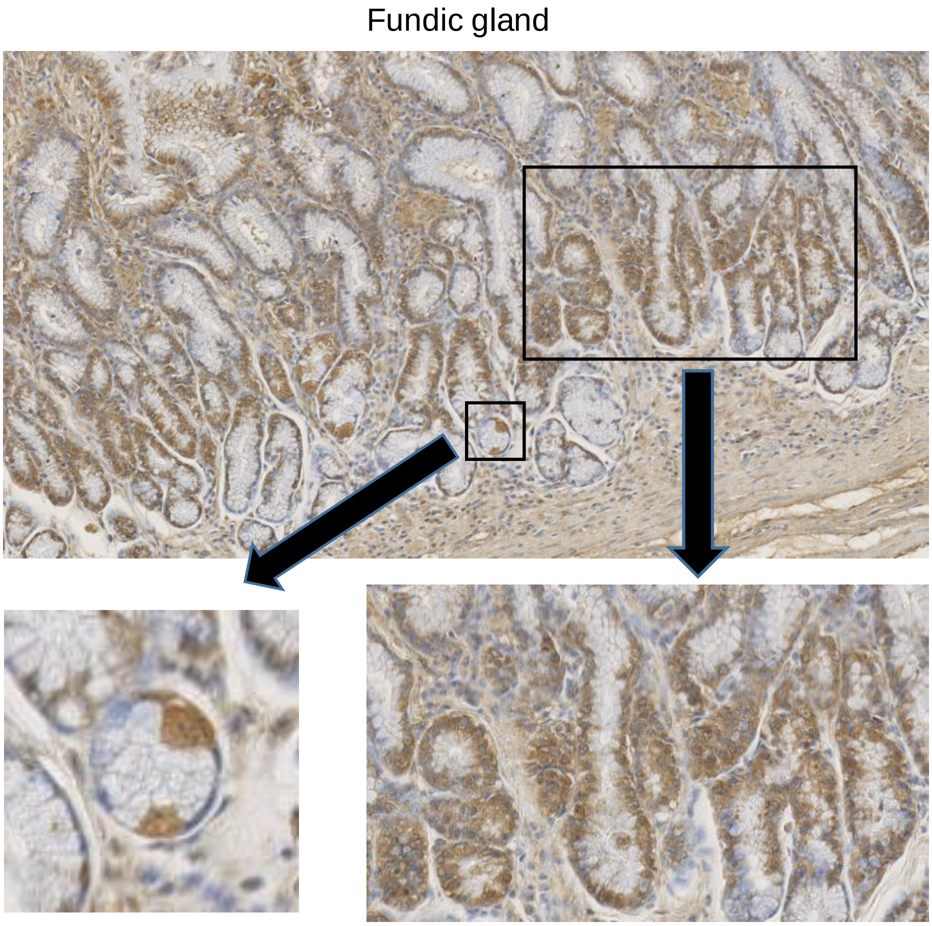

Supplementary Figure 10
Immunostaining of active Rac/Cdc42, showing activation of both Rac members and Cdc42, in fundic gland is displayed.

Supplementary Figure 11

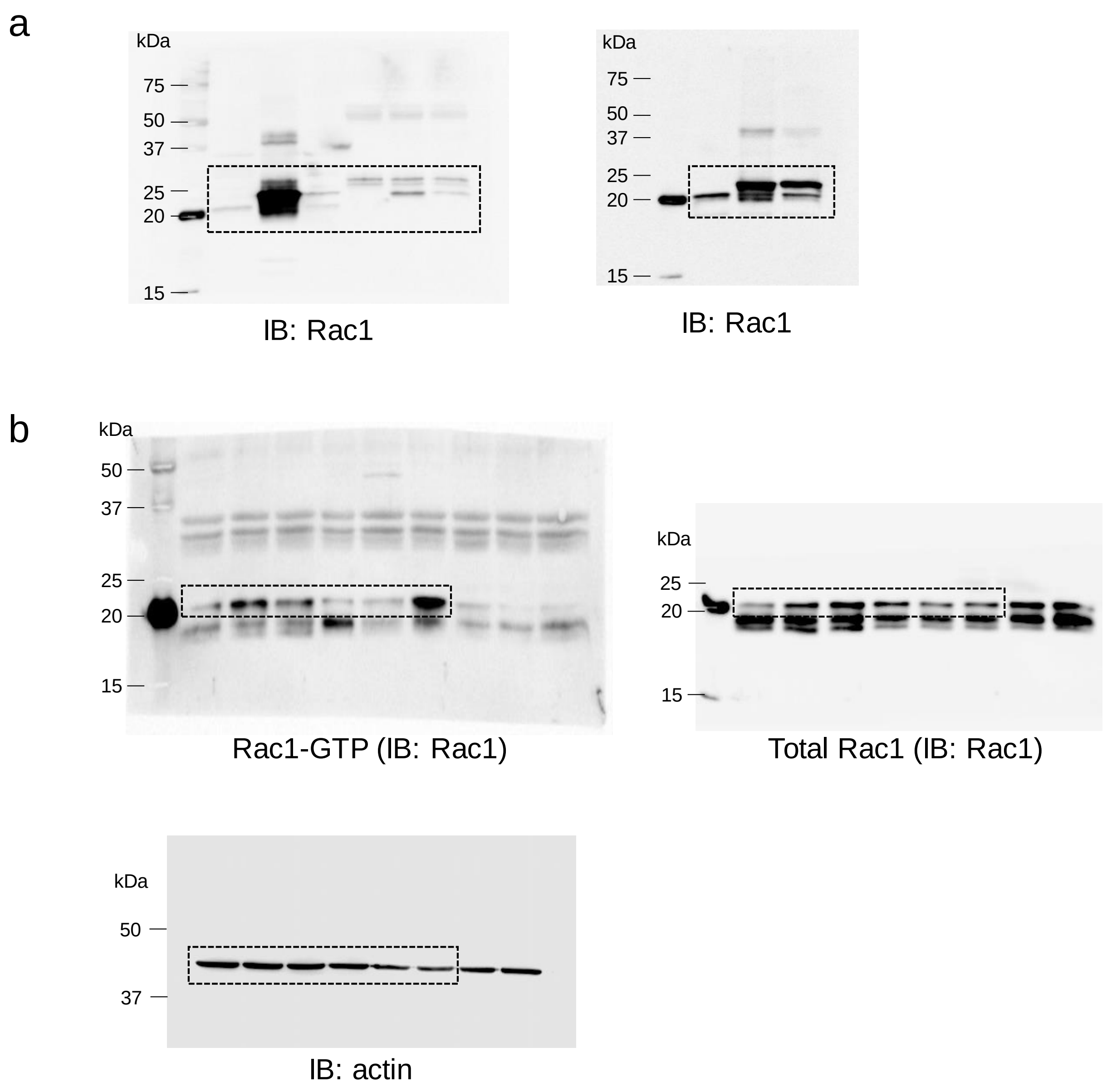

Supplementary Figure 11

Full length membrane data of immunoblotting for Fig. 1a (a) and Fig. 3a (b) with membrane edges visible. In (b), the membranes for total Rac1 and actin were cut prior to hybridization with each antibody. All images were taken with auto exposure time of a LAS4000 mini (GE Healthcare) and the contrast was adjusted with ImageQuant TL software (GE Healthcare) automatically.

Supplementary Figure 12

a

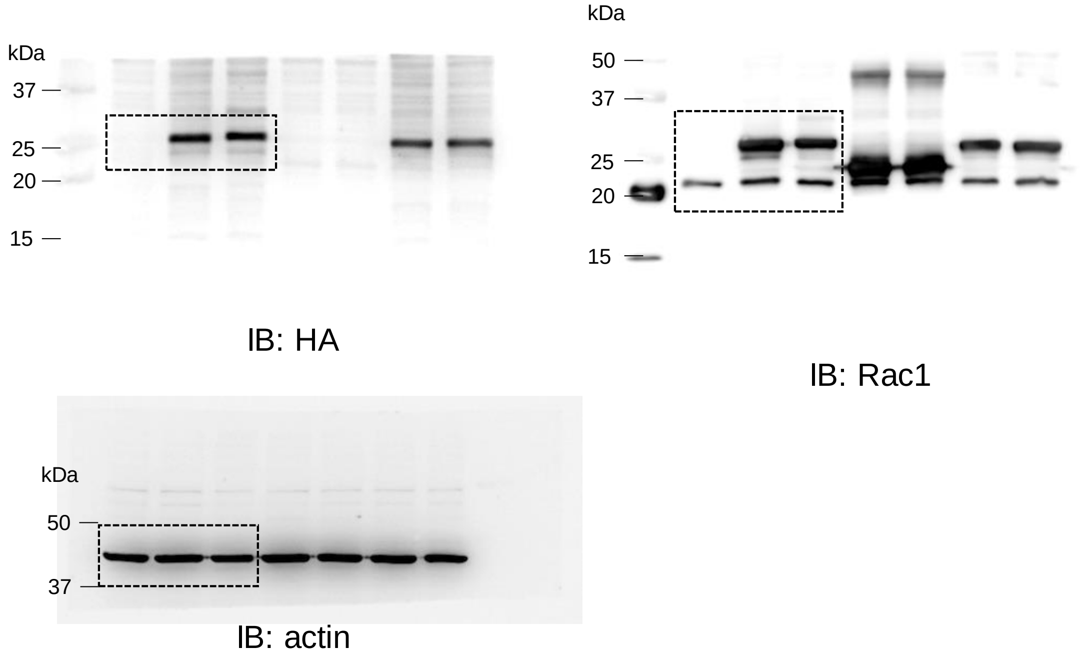

b

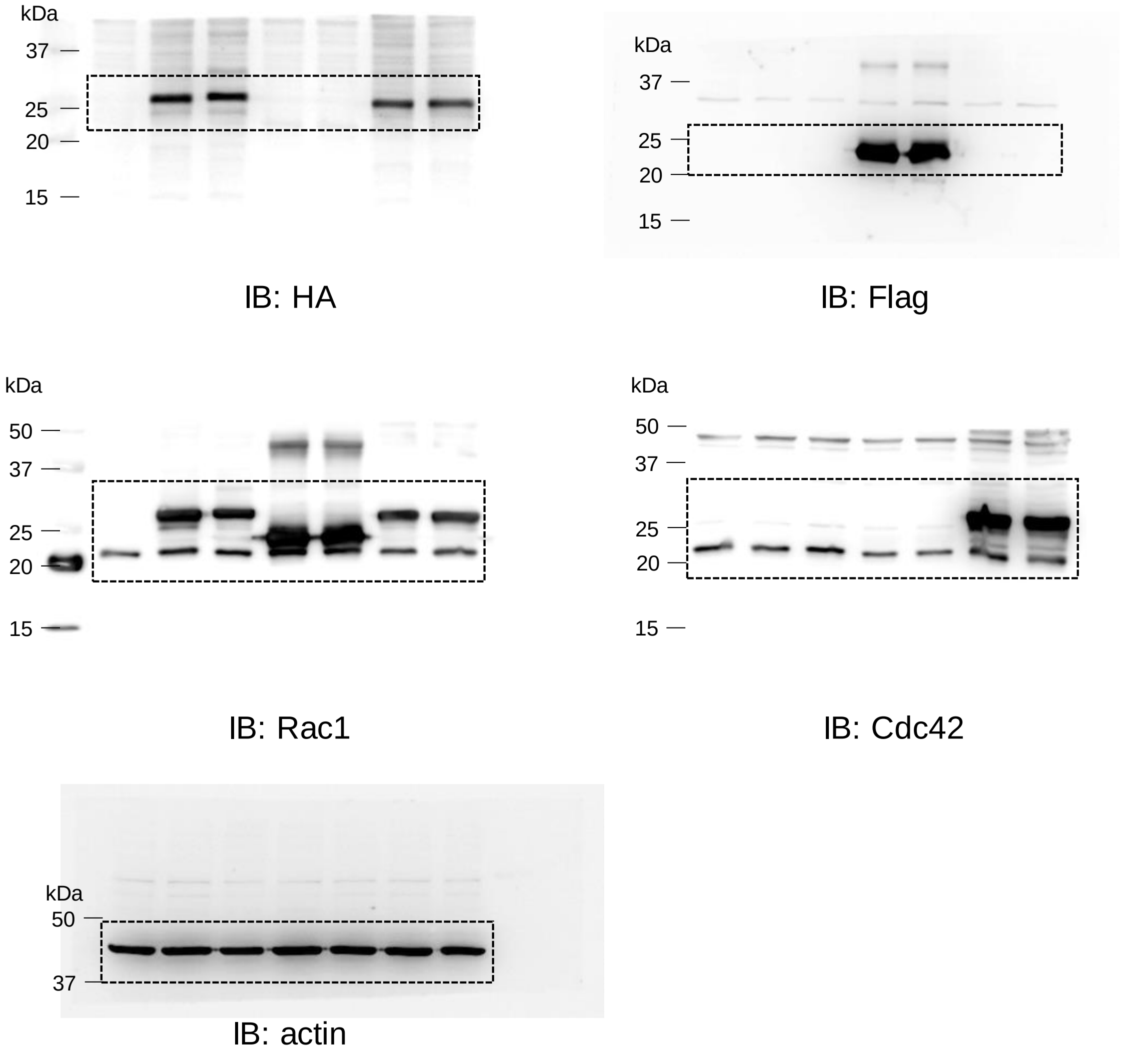

Supplementary Figure 12

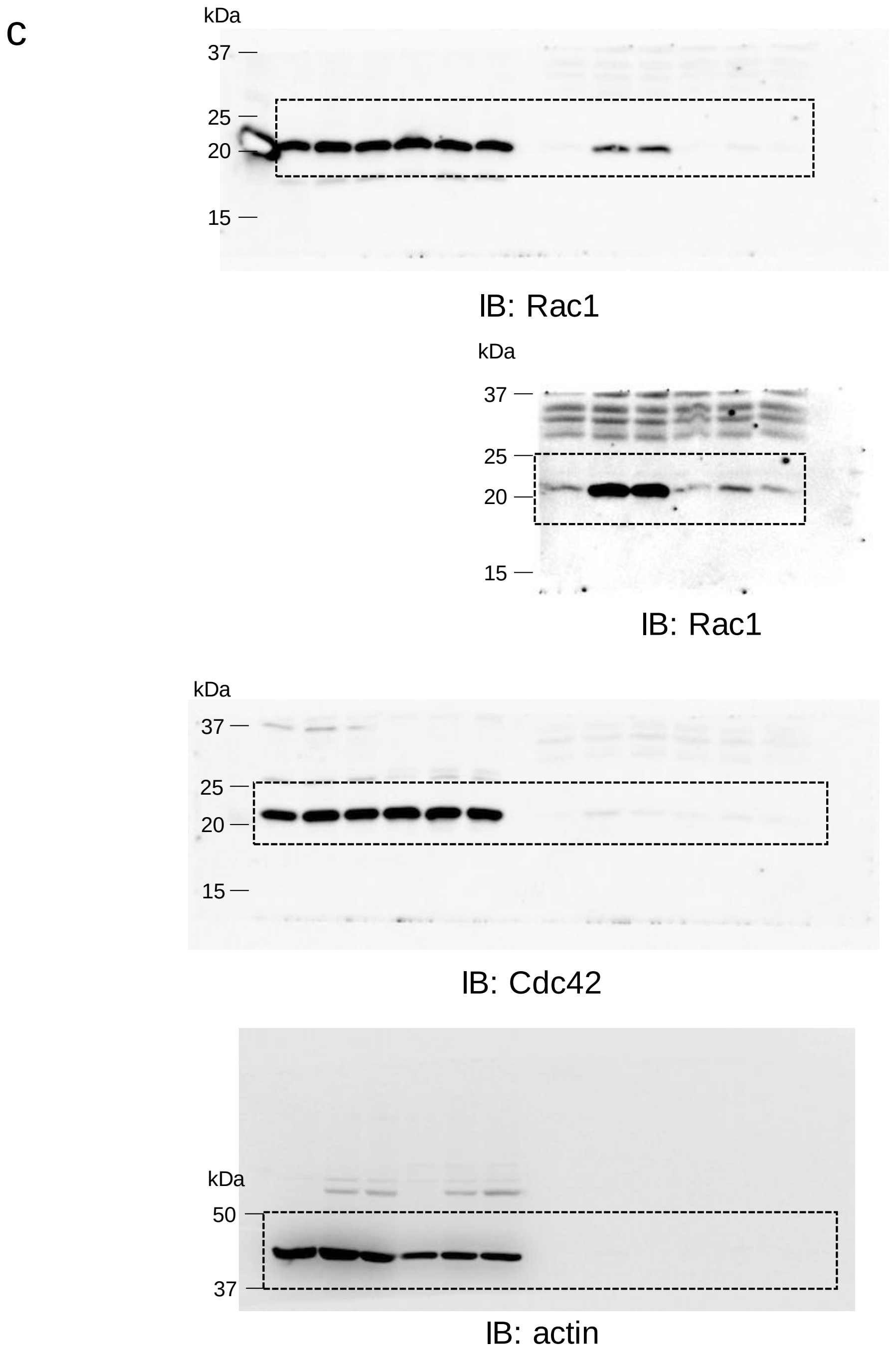

Supplementary Figure 12

Full length membrane data of immunoblotting for Supplementary Fig. S2a (a), Supplementary Fig. S4a (b), and Supplementary Fig. S5a (c) with membrane edges visible.

In (c) Rac1 immunoblotting, in order to efficiently detect activated Rac1 in the GST pull-down assay, the left membrane of the first panel (showing total cell lysate) was covered with aluminum foil, and the image was taken again and displayed on the second panel. All images were taken with auto exposure time of a LAS4000 mini (GE Healthcare) and the contrast was adjusted with ImageQuant TL software (GE Healthcare) automatically.

Supplementary Table 1

50 cases of human colon cancer FFPE tissues.

	Age Average	Sex (M:F)	Tumor location
Stage 0	80.7	1:2	A-colon: 2 cases<br>R-colon: 1 case
Stage Ⅰ	72.2	9:1	S-colon: 6 cases<br>T-colon: 2 cases<br>R-colon: 1 case<br>C, A-colon: 1 case
Stage Ⅱ	77.3	3:7	S-colon: 6 cases<br>T-colon: 1 case<br>A-colon: 2 cases<br>D-colon: 1 case
Stage ⅢA	72.9	4:6	S-colon: 6 cases<br>A-colon: 1 case<br>C, A-colon: 1 case<br>C-colon: 1 case<br>R-colon: 1 case
Stage ⅢB	73.3	2:5	S-colon: 6 cases<br>T-colon: 1 case
Stage IV	71.4	5:5	S-colon: 6 cases<br>C-colon: 1 case

Supplementary Table 2

Activation patterns of Rac and Cdc42 in 50 cases of colon cancer FFPE tissues.

Stage \ pattern	membrane	mixed (membrane+ diffuse)	diffuse	polarity	total
Stage 0				3	3
Stage Ⅰ		1	2	7	10
Stage Ⅱ	2		5	3	10
Stage ⅢA	2		5	3	10
Stage ⅢB			1	6	7
Stage Ⅳ	1		5	4	10

Supplementary Table 3

Rac/Cdc42 activity in primary S-colon cancer and metastatic lymph node

Stage	Case No.	S-colon tumor	Lymph node metastasis
Stage IIIA	Case 1	3+	3+
	Case 2	3+	3+
	Case 3	2+	1+
	Case 4	2+	N.D.
	Case 5	1+	3+
Stage IIIB	Case 1	3+	N.D.
	Case 2	1+	2+
	Case 3	2+	3+
Stage IV	Case 1	3+	3+
	Case 2	3+	N.D.
	Case 3	3+	3+
	Case 4	2+	3+
